# Current clinical opinion on surgical approaches and rehabilitation of hand flexor tendon injury—a questionnaire study

**DOI:** 10.3389/fmedt.2024.1269861

**Published:** 2024-02-15

**Authors:** Ruikang Xue, Jason Wong, Angela Imere, Heather King, Peter Clegg, Sarah Cartmell

**Affiliations:** ^1^ Department of Materials, Faculty of Science and Engineering, School of Natural Sciences, University of Manchester, Manchester, United Kingdom; ^2^ Division of Cell Matrix Biology & Regenerative Medicine, University of Manchester, Manchester, United Kingdom; ^3^ Department of Plastic & Reconstructive Surgery, Manchester Academic Health Science Centre, Manchester University NHS Foundation Trust, Manchester, United Kingdom; ^4^ The Henry Royce Institute, Royce Hub Building, The University of Manchester, Manchester, United Kingdom; ^5^ Addos Consulting Ltd, Winchester, United Kingdom; ^6^ Department and of Musculoskeletal and Ageing Science, Institute of Life Course and Medical Sciences, William Henry Duncan Building, University of Liverpool, Liverpool, United Kingdom; ^7^ MRC-Versus Arthritis Centre for Integrated Research in Musculoskeletal Ageing, William Henry Duncan Building, University of Liverpool, Liverpool, United Kingdom

**Keywords:** flexor tendon, repair, retrieval, survey, clinical opinion

## Abstract

The management of flexor tendon injury has seen many iterations over the years, but more substantial innovations in practice have been sadly lacking. The aim of this study was to investigate the current practice of flexor tendon injury management, and variation in practice from the previous reports, most troublesome complications, and whether there was a clinical interest in potential innovative tendon repair technologies. An online survey was distributed via the British Society for Surgery of the Hand (BSSH) and a total of 132 responses were collected anonymously. Results showed that although most surgeons followed the current medical recommendation based on the literature, a significant number of surgeons still employed more conventional treatments in clinic, such as general anesthesia, ineffective tendon retrieval techniques, and passive rehabilitation. Complications including adhesion formation and re-rupture remained persistent. The interest in new approaches such as use of minimally invasive instruments, biodegradable materials and additive manufactured devices was not strong, however the surgeons were potentially open to more effective and economic solutions.

## 1 Introduction

Flexor tendon injuries are one of the most common ailments in hand surgery that can lead to long-term disability and significant negative social and economic impact (1, 2). Despite a wealth of research in the field, management of hand flexor tendon injury remains inconsistent in approaches and outcomes (3). Current evidence revealed several beneficial development and change in practice in the field, including the use of wide-awake local anesthesia no tourniquet (WALANT) technique (4), updated methods of retrieving the retracted tendon stump (5–8), change in tendon repair approaches (9), the use of early active mobilization in post-surgery rehabilitation (10).

Use of endoscope and other minimally invasive surgical instrument has been described in the literature for the retrieval of the retracted tendon stump (11) and flexor tendon repair (12). With advances in the surgical instrumentation, minimally invasive surgeries have been widely used in other tendon repair such as Achilles tendon to deliver beneficial outcome (13). Development in tissue engineering, biomaterials and additive manufacturing has further potential in improving tendon repair outcome (14, 15).

In this study, we distributed an online questionnaire to hand surgeons via the British Society for Surgery of the Hand (BSSH) to survey the current clinical practice on hand flexor tendon injury management, including anesthesia, tendon retrieval, tendon repair, post-surgery rehabilitation, operative time and complications. Firstly, we aimed to assess the impact of the current medical research evidence on clinical practice, and identify if there is any trend from previous survey studies. Finally, we aimed to gather surgeons' opinions on the adapting potential novel solutions to treat hand flexor tendon injury enabled by minimally invasive instrumentation, biomaterials and additive manufacturing.

## 2 Methods

A 26-item online survey was developed, containing 22 single-answer multiple-choice questions and 4 open-ended questions (see Supplementary Table S1). “Other, please specify” option was included in all the multiple-choice questions to improve study flexibility. The first 3 open-ended questions were follow-on questions that were designed to enable the respondents to add any additional comments on tendon retrieval technique used, challenges in tendon retrieval, and estimated complication rate of the most common complication the respondents mentioned. The last open question was designed to enable the respondents to add any additional comments on any aspect of flexor tendon injury management. The survey covered a number of aspects of hand tendon injury management, including anesthesia, tendon retrieval, primary tendon repair, peripheral (i.e., epitendinous) repair, post-surgery rehabilitation, average operation time, complications, as well as opinions on some potential solutions. Demographics of respondents including gender, age, ethnic background, experience in hand specialty, type of their surgery were also collected. The survey was peer reviewed by the authors and in consultation with external hand surgeons, and subsequently approved by the University of Manchester Research Ethics Committee (2019-7707-11796).

The survey was electronically delivered to surgeons through the British Society for Surgery of the Hand (BSSH) communication channels (email and Twitter) to BSSH members and associates.

The results remained anonymous and were analyzed using GraphPad Prism 8.4.3 (GraphPad Software, USA) where, for each question, the total number of answers (*N*) was obtained, and the percentage of each option was calculated. The comments from the open-ended questions were categorized and the percentage of each category was calculated.

## 3 Results

A total of 132 individual survey responses were completed. The full demographic information collected can be found in Supplementary Table S2. Most respondents had been in hand specialty for over 11 years (48%, 61/128), with 23% (30/128) being in hand specialty for 8–11 years, 22% (28/128) for 4–7 years and 7% (9/128) for less than 4 years. The majority of respondents (71%, 91/129) were working in National Health Service (NHS) in the UK whereas 4% (5/129) respondents were working only in private surgery; the rest of the respondents (26%, 33/129) were involved in both NHS and private surgery.

**Figure 1:**
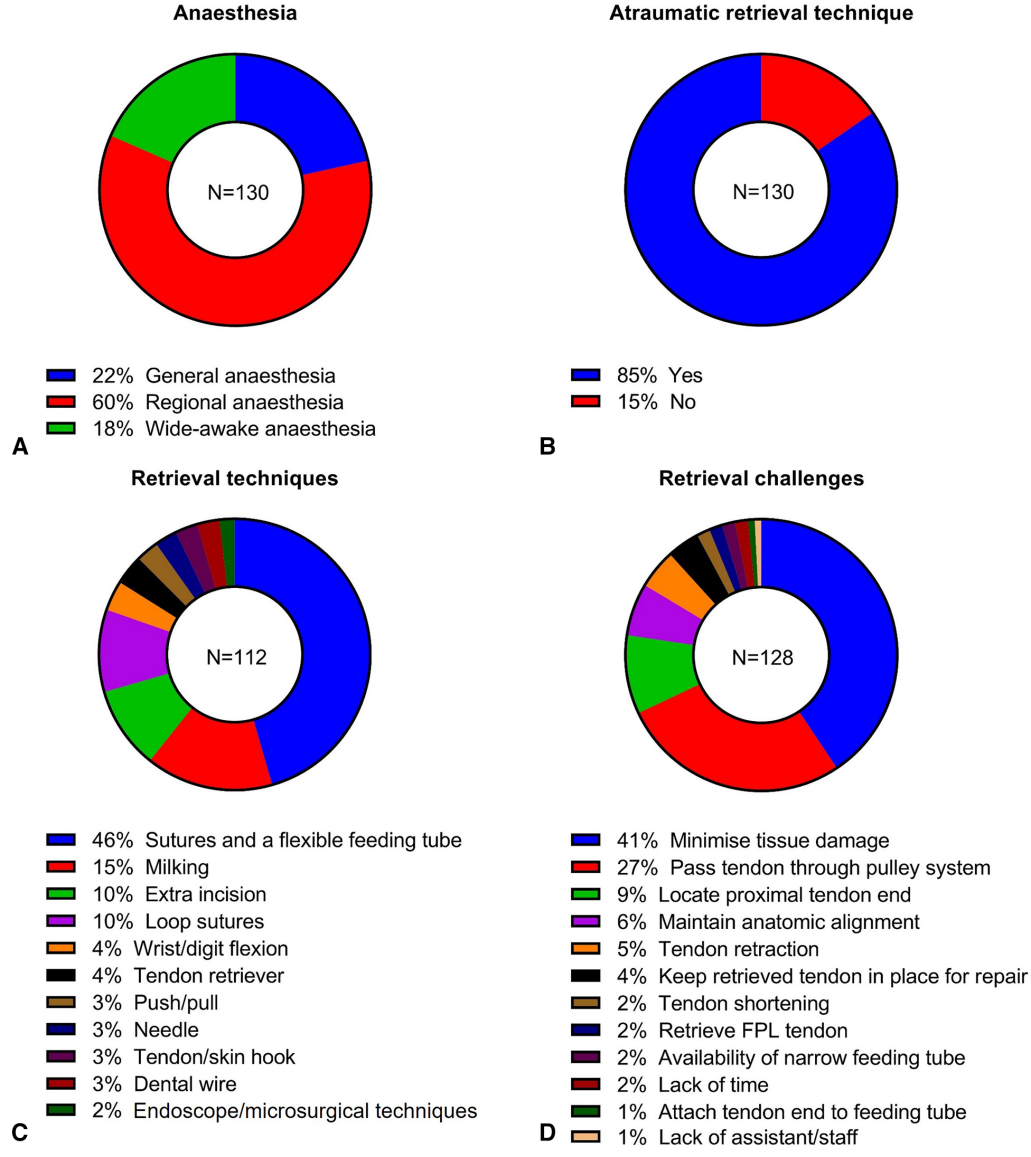
Results of anesthesia used for flexor tendon repair (**A**), use of atraumatic tendon retrieval (**B**), tendon retrieval techniques (**C**) and challenges in tendon retrieval (**D**).

In terms of anesthesia used for flexor tendon repair, most respondents preferred regional anesthesia (60%, 78/130), whilst others favored the use of general anesthesia (22%, 28/130) or WALANT (18%, 24/130, Figure 1A).

The majority of the respondents used atraumatic tendon retrieval techniques (85%, 110/130) if there was retraction of the proximal end of the tendon stump (Figure 1B). Most respondents described using sutures along with either a small gauge flexible feeding tube or equivalent material for tendon retrieval (46%, 51/112, Figure 1C). Other methods mentioned include milking (15%, 17/112), creating extra incisions (10%, 11/112), use of loop sutures (10%, 11/112), wrist or digit flexion (4%, 4/112), use of tendon retrievers (4%, 4/112), push and pull method (3%, 3/112), use of needle (3%, 3/112), use of tendon or skin hook (3%, 3/112), use of dental wire (3%, 3/112) and use of endoscope or microsurgical techniques (2%, 2/112).

Forty-one percent (52/128) respondents considered minimizing tissue damage as the key challenge in flexor tendon retrieval (Figure 1D); preservation of tendon sheath and pulley structure, and maintaining the tendon stump integrity were specifically mentioned. Furthermore, passing tendon stump through the pulley system was frequently mentioned (27%, 35/128), followed by locating proximal tendon end (9%, 12/128), maintain anatomic alignment to avoid decussation (6%, 8/128), tendon retraction (5%, 6/128) and keeping retrieved tendon ends apposed during surgical repair (4%, 5/128). Five other challenges were also mentioned once or twice by the respondents – tendon shortening, retrieval of *flexor pollicis longus* (FPL) tendon, availability of narrow feeding tube, lack of time, attaching the tendon to the feeding tube and lack of a surgical assistant.

**Figure 2:**
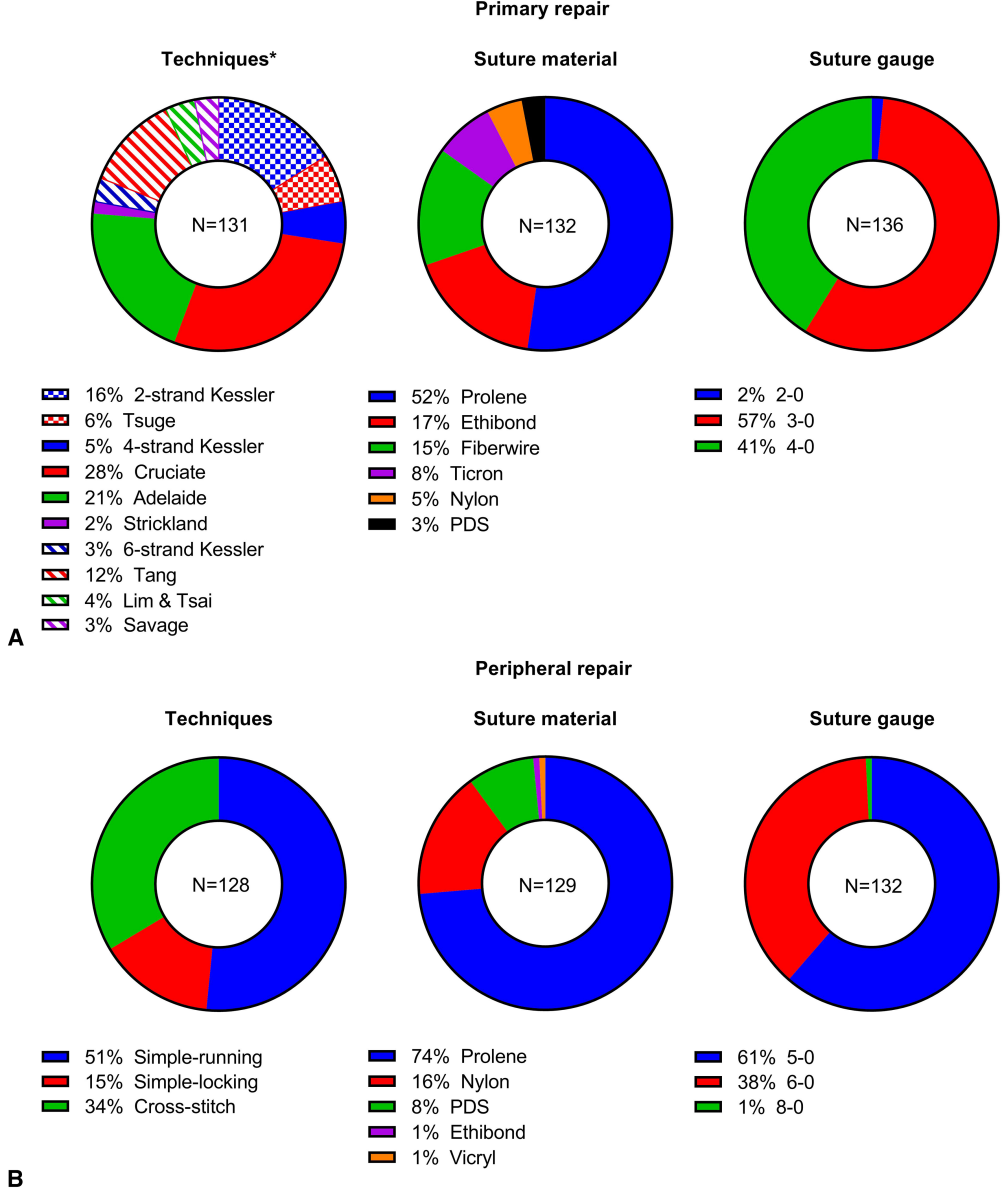
Results of repair techniques, suture materials and suture gauge on primary (**A**) and peripheral tendon repair (**B**). *Checked – 2 strand repairs, solid – 4 strand repairs and hatched – 6 strand repairs.

For primary repair, a wide range of techniques were used, among which the Cruciate suture pattern (28%, 37/131) was the most popular (Figure 2A). Four-strand repairs (56%, 76/131) were preferred by the respondents to either the 2-strand (22%, 29/131) or 6-strand repairs (22%, 29/131). Prolene (52%, 69/132) suture material was most frequently used, followed by Ethibond (17%, 23/132), Fiberwire (15%, 20/132), Ticron (8%, 10/132), Nylon (5%, 6/132) and PDS (3%, 4/132). For suture gauge, 3-0 (57%, 78/136) and 4-0 (41%, 56/136) sutures were used by most respondents whereas 2-0 gauge was only mentioned twice (2%, 2/136).

For the peripheral repair, simple running suture technique (51%, 66/128) was most preferred by the respondents, followed by cross-stitch (34%, 43/128) and simple locking (15%, 19/128, Figure 2B). Noticeably, under cross-stitch technique, Silfverskiöld repair was mentioned 27 times (21%, 27/128). Like primary repair, Prolene (74%, 95/129) was the most frequently used suture material; other materials reported include Nylon (16%, 21/129), PDS (8%, 11/129), Ethibond (1%, 1/129) and Vicryl (1%, 1/129). Sutures with 5-0 (61%, 81/132) and 6-0 gauge (38%, 50/132) were used by most respondents with only 1 individual using 8-0 gauge (1%, 1/132).

**Figure 3:**
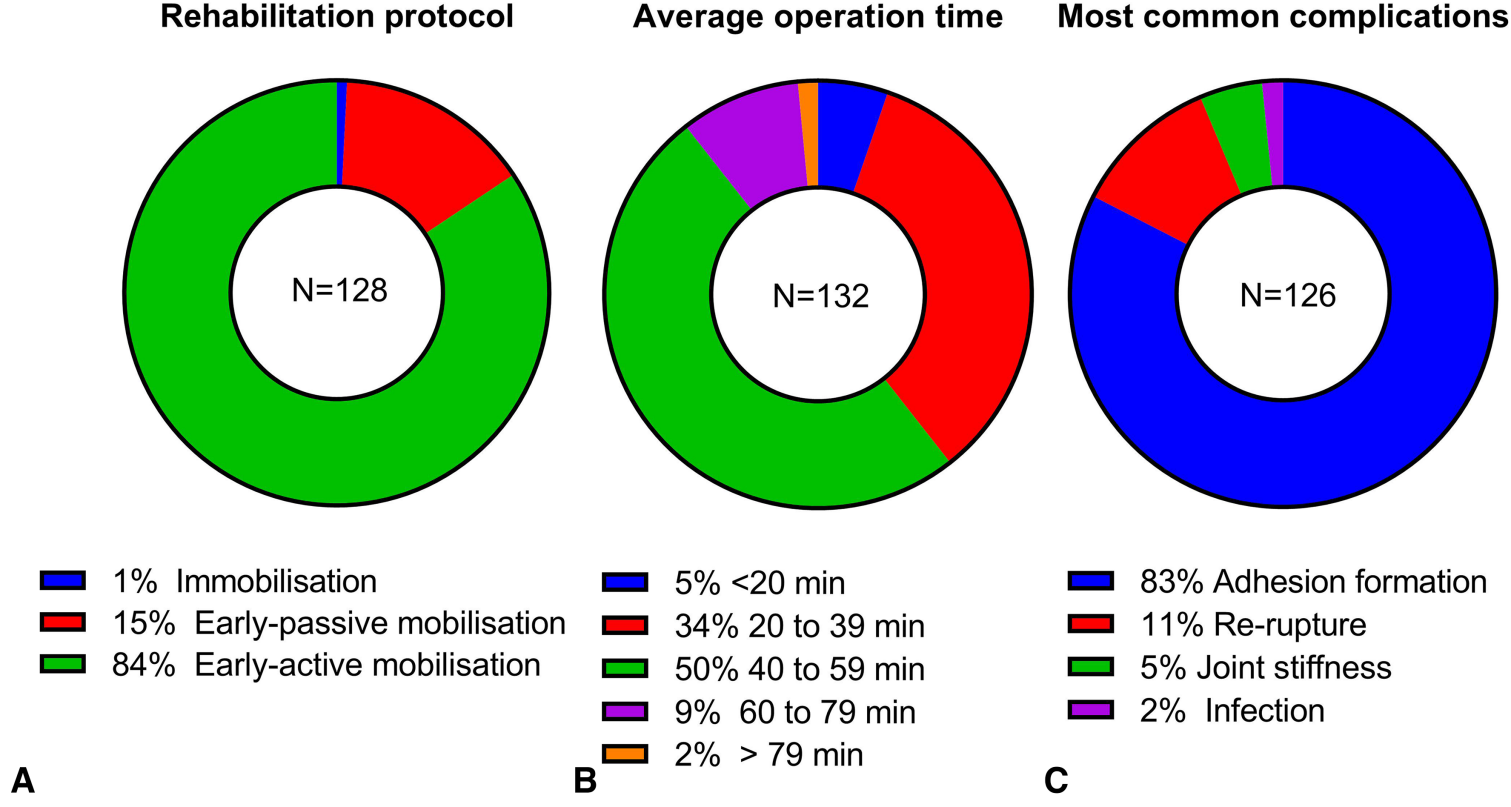
Results of rehabilitation protocol (**A**), average hand tendon repair operation time (**B**), and most common complications (**C**).

In terms of rehabilitation protocol, most respondents would support early active mobilization (84%, 108/128, Figure 3A). On the other hand, early passive mobilization protocol (15%, 19/128) was not uncommon. Immobilization was selected by one respondent (1%, 1/128).

On average, the majority the respondents (89%, 118/132) spent less than 1 h on hand tendon repair operations with 5% respondents under 20 min (7/132), 34% from 20 to 39 min (45/132), and 50% from 40 to 59 min (66/132). However, 12 respondents (9%) selected 60–79 min and 2 respondents (2%) spent over 79 min (Figure 3B).

Adhesion formation (83%, 104/126) was identified as the most common complication of flexor tendon repair by the majority of the respondents (Figure 3C). Other respondents considered re-rupture (11%, 14/126), joint stiffness (5%, 6/126) or infection (2%, 2/126) as the most common complication. Comments from the respondents on the estimated complication rate indicated that most patients would get some adhesion formation and complicated adhesion accounted for 5%–20% of the patients. For the estimated percentage of re-rupture, 45% (5/11) respondents indicated under 5% and another 45% (5/11) responses indicated 5%–10%, whereas one respondent answered 15%. Only 1 respondent commented on the complication rate of joint stiffness – “majority cases”. The estimated infection rate was below 5% from 2 respondents.

**Figure 4:**
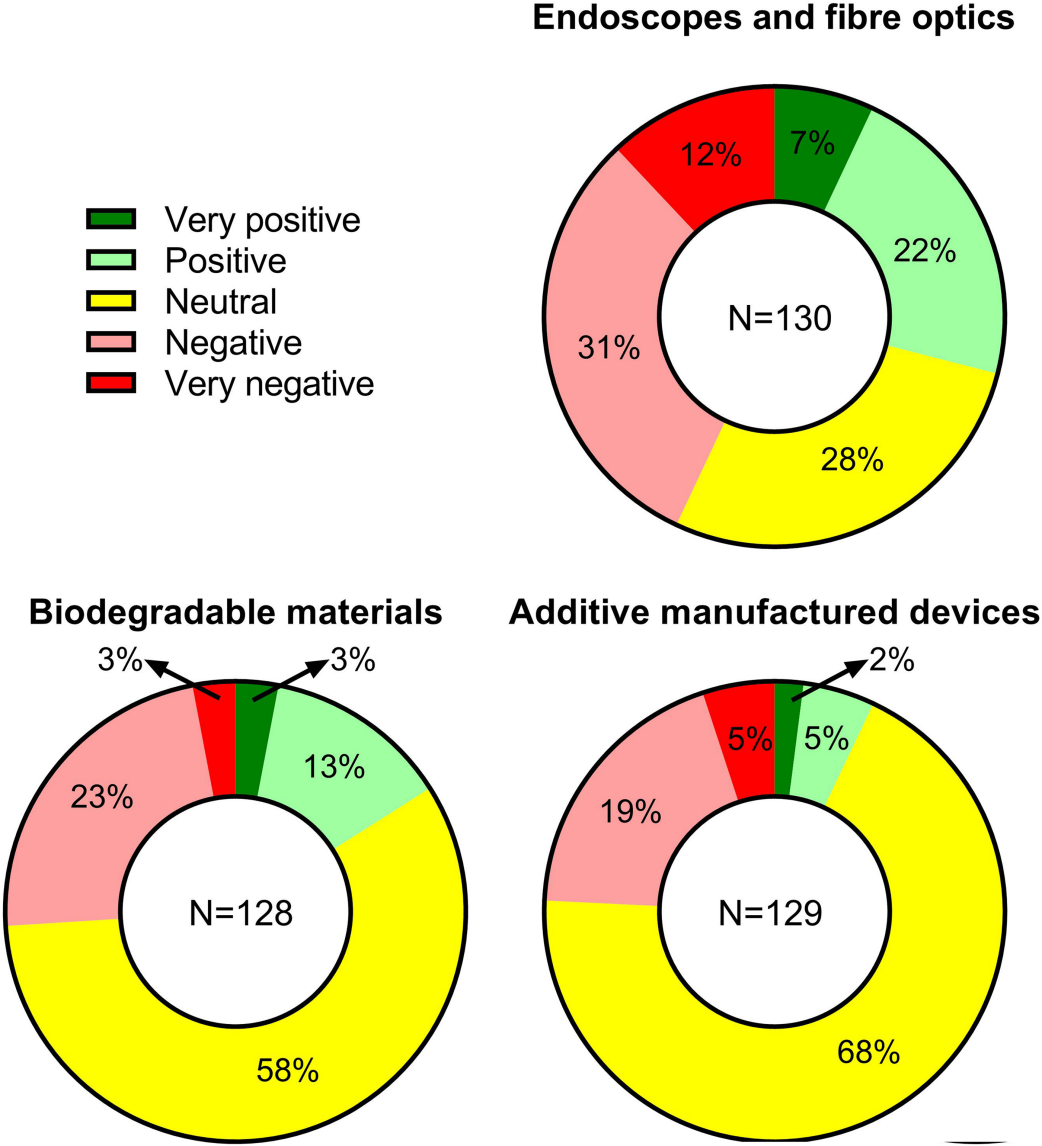
Clinical opinions on new approaches to treat hand flexor tendon injuries.

In Figure 4, questions regarding clinical opinions on novel approaches to tendon repair revealed that only 29% (38/130) of the respondents supported the use of endoscopes or fiber optic technology, whereas a significant portion of the respondents (43%, 55/130) reacted negatively. In terms of the use of degradable biomaterials, the majority of respondents had a neutral response (58%, 74/128); from the remaining participants, more negative responds (26%, 34/128) were found compared to positive ones (16%, 20/128). Similarly, for the use of additive manufacturing, neutral responses were received by most participants (68%, 88/129), followed by negative (24%, 32/129) and positive (7%, 9/129) responses. Interestingly, in contrary to results from the multiple-choice questions, six out of eight comments on the topic from the open-ended question indicated that there is still a room for more expensive novel approaches provided they are simple to use and can provide better outcome.

## 4 Discussion

Three previous questionnaire studies on hand flexor tendon injury management were identified from the literature. Healy et al. surveyed 22 consultant surgeons in Ireland in 2007 (16); Rudge et al. surveyed 39 hand units in the UK and Ireland with responses from their lead consultant surgeon (17); and Gibson et al. surveyed 410 individual surgeons with varied experience in the USA (18). In comparison, this study surveyed 132 individual surgeons with varied experience in the UK, which was the highest in the UK. The comparison between current medical evidence, previous survey findings and this this work is summarized in Table 1.

**Table 1:** Comparison between current evidence, previous questionnaire studies and the current work on aspects of flexor tendon injury management investigated.

	Current evidence	Previous survey study			Current work
		Healy et al. (16)	Rudge et al. (17)	Gibson et al. (18)	
Respondent demographics		22 Individuals, Consultant surgeons, Ireland	39 Hand units, Lead consultant surgeon, UK and Ireland	410 Individuals, Varied experience, USA	132 Individuals, Varied experience, UK
Anaesthesia	WALANT offers better patient education, intra-operative assessment, reduced risk of Covid transmission, and economic benefits to patient and hospital (4, 19, 20).			20% performed WALANT, 45% of which preferred it	18% WALANT, majority (60%) preferred general anaesthesia
Retrieval of retracted tendon stump	A mix of retrieval methods are used. Atraumatic approach should be used where possible as minimizing tissue damage is the highest priority during tendon retrieval (1, 11). Recent techniques advocated the use of sutures, a flexible tube and a proximal palmar incision (5–8).				85% used atraumatic approach, 11 different techniques reported, Use of sutures and a flexible feeding tube (46%) most common approach, but “milking” still common (15%). Minimising tissue damage (41%) most common challenge, but numerous other challenges also reported.
Primary repair	A mix of repair methods are used, Multi-strand repair showed improved strength and rupture rate in biomechanical studies but its clinical evidence is less clear (21–23). Braided polyester sutures showed better strength than monofilament Prolene sutures (3, 18).	Two most popular repair methods			
		Kessler (68%), Adelaide (23%)	Kessler (64%), Strickland (18%)	Kessler (42%), Cruciate (26%)	Cruciate (28%), Adelaide (21%)
		2-strand vs. multi-strand methods			
		2-strand (64%), multi-strand (32%)	2-strand (36%), multi-strand (64%)	2-strand (6%), multi-strand (94%)	2-strand (16%), multi-strand (84%)
		Suture materials: Prolene vs. braided polyester (Fibrewire, Ethibond)			
		Prolene (45%), braided polyester (36%)	Prolene (64%), braided polyester (34%)	Prolene (8%), braided polyester (54%)	Prolene (52%), braided polyester (32%)
		Suture gauge			
		3-0 (50%), 4-0 (50%)	3-0 (82%), 4-0 (18%)	3-0 (52%), 4-0 (47%)	3-0 (57%), 4-0 (41%)
Peripheral repair	Cross-stitch offers higher mechanical strength than simple running and locking (24, 25).	Two most popular repair methods			
		Simple running (73%)		97% used peripheral repair	Simple running (51%), Cross-stitch (34%)
		Two most popular suture material			
		Prolene (64%), Nylon (27%)	Prolene (82%), Nylon (18%)		Prolene (74%), Nylon (16%)
		Suture gauge			
			5-0 (28%), 6-0 (72%)		5-0 (61%), 6-0 (38%)
Rehabilitation	EAM offers improved functional outcome and economic benefits with a trade-off for slight increase of re-rupture rates (26, 27).			EAM (51%), PEM (49%)	EAM (84%), EPM (15%)
Operation time	NHS UK indicates 45–60 min for a simple flexor tendon repair (28).				Most common 40–59 min (50%), and 20–39 min (34%)
Complications	4% re-rupture, 4% adhesion formation (29).				Most common (83%) with estimated rate of 5%–20% for complicated adhesion. Re-rupture (11%) with estimated rate of 5%–10% from 10/11 respondents.

WALANT, wide-awake local anesthesia no tourniquet; EAM, early active mobilization; EPM, early passive mobilization; NHS, national health service.

Traditional anesthesia approaches including general and regional anesthesia along with tourniquet application are routinely practiced in hand flexor tendon repair. In the past two decades, WALANT that injects lidocaine with epinephrine directly to the operative site, which enables patients to remain conscious during the operation, gained increasing popularity (30, 31). It showed comparative flexor tendon repair outcome to general and regional anesthesia (32), and a number of additional advantages, including intraoperative assessment of tendon repair, reduction in operation time and surgery cost, faster patient discharge, better patient education (4, 19). Gibson survey reported that 20% respondents performed WALANT in the past, and 45% of which preferred it when situation allows. In comparison, this study showed an increase in preference of using WALANT (18%) (18). Following the COVID-19 pandemic, numerous centers reported a shift towards WALANT due to its reduced risk to healthcare professionals from contracting COVID-19 by removing the use of aerosol-generating traditional anesthesia (20, 33), which is expected to cause further increase in the use of WALANT.

In zone II flexor tendon injury, retrieval of retracted proximal tendon stump can be problematic due to the presence of the relatively inelastic tendon sheath; trauma created during retrieval can lead to poor functional outcome and needs to be minimized. A plethora of tendon retrieval methods were published in literature (1, 11). Agreed with the current evidence, in this study, most respondents (85%) supported atraumatic tendon retrieval, and reported a variety of retrieval techniques. Recent development of flexor tendon retrieval techniques advocated the use of sutures, a flexible tube and a proximal palmar incision, in which the tendon stump was connected to the flexible tube and successfully retrieved through the tendon sheath (5–8). Most respondents (45%) mentioned the use of sutures and a flexible feeding tube, and some respondents (10%) also mentioned the creation of an extra incision. However, a number of the respondents (15%) still chose “Milking” in which the proximal tendon stump was milked down through the tendon sheath despite its low success rate (61%) (11). Supported by 10% of the respondents, looped sutures were able to retrieve the proximal stump through tendon sheath without the need of a feeding tube (34). It is worth noting that almost 1 in 5 respondents used other retrieval techniques, showing the diversity in tendon retrieval methods in practice. In alignment with the current evidence, minimizing tissue damage (commented by 41% respondents) is most challenging and is the highest priority during tendon retrieval (1). However, comments received from this work considered a variety of other factors as most common challenges, including passing tendon through pulley system (27%) that has an important role in translating force from muscles to phalanges (35), difficulty in locating the retracted tendon (9%) that sometimes requires multiple incisions (1), maintaining the anatomic alignment of retrieved tendons (6%) that can affect the mechanical efficiency of figure flexion (36), and eight additional items, each of which commented by less than 5% of the respondents. No information on flexor tendon retrieval was reported in the previous survey studies.

An ideal primary flexor tendon repair aims to provide strong repair strength to minimize risk of re-rupture with minimal bulkiness that can interfere with tendon gliding and ultimately result in adhesion formation (21). In the past decade, multi-strand core sutures (4-strand or more) with 3-0 or 4-0 sutures gained increasing popularity across the globe (9, 21). Laboratory biomechanical studies showed superior repair strength and lower re-rupture rate of multi-strand repairs; however, clinical studies showed no significance in overall re-rupture rate despite that re-rupture occurred later (after 4 weeks) in multi-strand repairs compared to 2-strand repairs (22, 23). Thinner sutures reduced tissue bulk and minimized tissue trauma whereas thicker sutures improved repair strength and ease of handling. 78% of respondents followed the current trend of using multi-strand core sutures (78%) and the majority of them (98%) used 3-0 or 4-0 sutures. 4-strand Cruciate and Adelaide repair techniques were used by almost half (49%) of the respondents, which was likely due to their good repair strength and ease of performance previously reported (37, 38). In terms of the suture materials, newer braided polyester sutures such as Fiberwire and Ethibond have been shown to have superior mechanical properties than monofilament sutures such as Prolene and Nylon (3, 18). However, Prolene (52%) was still the choice from the most respondents from the current survey, followed by braided polyesters (32%), and 16% other materials (Ticron, Nylon and PDS). Compared to previous surveys in the UK and Ireland (16, 17), an increased use of multi-strand core sutures and Cruciate suturing was observed, whereas there was a limited change in suture material; interestingly, more 3-0 sutures (82%) were reported despite of an uptake of multi-strand (64%) repair in Rudge's study. In contrast, Gibson's survey indicated that more surgeons in the US supported the use of multi-strand core (94%), Kessler type repair (42%) and braided polyester materials (52%) (18).

Peripheral repairs are often used in combination with primary core suture repairs to improve repair strength, prevent fraying and decrease friction (24). In vitro biomechanical study revealed that Cross-stitch had higher mechanical strength than simple-running and simple-locking techniques (24, 25). Despite inferior mechanical strength, simple-running technique was identified by more than half of respondents (51%), which was likely due to its ease of use (24). Compared to previous surveys (16, 17), an increased use of Cross-stitch was identified, and there was not much change in suture materials as Prolene and Nylon remained the most popular choices; more 5-0 sutures were used in the current study compared to Rudge's.

Since 1940s, flexor tendon rehabilitation has progressed from immobilization to early passive mobilization to early active mobilization (39). Recent studies showed that early active mobilization improved functional outcome including increased range of motion, reduced joint stiffness and adhesions, with a trade-off for slight increase of re-rupture rates (26, 27). Furthermore, early active mobilization is the least therapist dependent method, leading to additional economic benefits (23). The majority of the correspondents (84%) followed the current scientific evidence to use early active mobilization rehabilitation, higher than the percentage (51%) in Gibson's report.

The NHS in the UK estimated the average operative time for a simple flexor tendon repair to be between 45 and 60 min (28), which was supported by half of respondents. Interestingly, 39% respondents were faster than the NHS suggestion, whilst 11% respondents were slower. Tang et al. reported the surgery time for performing a primary repair with various suturing techniques, ranging from 6.2 to 13.5 min (40). Apart from primary tendon repair, incision for tendon access, tendon retrieval, peripheral repair and pulley reconstruction can also affect the total operative time in flexor tendon surgery (1).

Meta-analysis on complications of flexor tendon repair from Dy et al. revealed a 6% average rate of re-operation, 4% of adhesion formation and 4% of re-rupture (29). Adhesion formation is frequently observed after surgery and complicated adhesions need surgical tenolysis; the rate of re-rupture has continuously decreased in the past decade but re-rupture still remains a persistent complication (41). Those two complications were mentioned by 94% of the respondents as the most common complications, with the their estimated rate ranging from 5% to 20% for complicated adhesions, and 5%–10% for re-rupture. Joint stiffness (5%) and infection (2%) were also mentioned by some respondents. Joint stiffness is common, but it normally improves with time through daily hand use; infection after flexor tendon repair is rare, which is most likely caused by contamination during initial trauma (41, 42). The functional outcome was reported to be over 80% with overall excellent and good recovery of functionality in recent years (41).

Minimally invasive instrumentation (e.g., endoscopes, fiber optics, microsurgical tools) has evolved rapidly and it can benefit hand tendon repair (43, 44). Recently, Kucukguven et al. employed an endoscope and 1 mm flexible forceps to atraumatically retrieve retracted tendons, showing significantly shorter operative duration, better pain score and higher total range of active motion in 11 patients compared to traditional retrieval methods (44). Biodegradable materials and tissue engineering are other ways to reduce flexor tendon repair complications since it was shown to enhance tendon healing and regeneration, leading to improved functional outcomes (45). Also, our previous studies indicated that suture repairs produced high stress regions and acellular zones on the tendon, which potentially contributed to early tendon failure (46, 47). Additive manufacturing and barbed connecting devices may provide unique solutions in reducing acellular zones and improving tendon repair (48, 49). This work revealed that the surgeons' interests in new approaches such as endoscopes, biodegradable materials and additive manufactured devices were not strong with most responses being neutral or negative. However, it was mentioned by several clinicians that there was still room for improvement in the field and approaches to reduce complications, improve functional outcome and shorten surgery time were still welcome.

As with most surveys, this study may have potential bias caused by incomplete sampling and underrepresentation of the non-responders (50, 51). Specifically, the response rate was not available due to the unknown number of total recipients of the survey. Also, this study did not take pediatric patients into consideration, which could provide more comprehensive knowledge on the current practice. Furthermore, geographical data was not collected although the majority of the respondents were believed to be in the UK because the questionnaire was distributed via the BSSH communications to its members and associates. Last but not least, some specific technical aspects of tendon repairs such as flexor digitorum superficialis tendon repair, A2/A4 pulley release, tendon sheath repair, comparison between delayed and primary repairs were not included in the survey, which could lead to enhancement of the impact of the publication.

In conclusion, this study revealed an increased number of surgeons followed the current medical evidence on flexor tendon injury management in the UK. However, a portion of surgeons still practiced suboptimal solutions, including costly general anesthesia, traumatic and ineffective tendon retrieval techniques, traditional two-strand repairs with monofilament suture materials, and passive rehabilitation protocols. Flexor tendon repair complications such as adhesion formation and re-rupture remained persistent. Variation in practice between surgeons from the UK and Ireland, and those from the US suggested that surgeons in the US were able to adopt new technology more quickly into clinic. Last but not least, in general, clinical interest in use of minimally invasive instruments, new biomaterials and additive manufactured devices was not strong; however, novel approaches that can improve repair outcome or provide economic benefits or both were welcomed by a portion of respondents.

## Data Availability

The original contributions presented in the study are included in the article/**Supplementary Material**, further inquiries can be directed to the corresponding author.
