# Supplementary material for: Current clinical opinion on surgical approaches and rehabilitation of hand flexor tendon injury—a questionnaire study
Source: Front Med Technol. 2024 Feb 15;6:1269861. doi: 10.3389/fmedt.2024.1269861 (PMC10902169; doi:10.3389/fmedt.2024.1269861)
Supplement: Supplementary file 1 [file Table1.docx]

Supplementary Material

# Supplementary Figures and Tables

Supplementary Table 1. List of questions in the questionnaire. NHS: National Health Service in the UK.

| **1. Which anaesthetic do you prefer when performing flexor tendon repair?** | | | | | | | | | | | | | | | | |
| --- | --- | --- | --- | --- | --- | --- | --- | --- | --- | --- | --- | --- | --- | --- | --- | --- |
| General anaesthesia | | | Regional anaesthesia | | | | | Wide-awake anaesthesia | | | | | | Other, please specify | | |
| **2. Do you use atraumatic tendon retrieval techniques?** | | | | | | | | | | | | | | | | |
| Yes | | | | | | | No | | | | | | | | | |
| **2.1 What tendon retrieval technique do you use?** | | | | | | | | | | | | | | | | |
|  | | | | | | | | | | | | | | | | |
| **3. What are the key challenges in tendon retrieval?** | | | | | | | | | | | | | | | | |
|  | | | | | | | | | | | | | | | | |
| **4. Your choice of primary repair technique.** | | | | | | | | | | | | | | | | |
| Kessler | | Savage | | | | | Strickland | | | Cruciate | | | | | Tang | |
| Other, please specify: | | | | | | | | | | | | | | | | |
| **5. Your choice of core suture material.** | | | | | | | | | | | | | | | | |
| Prolene | | Ethibond | | | | | Ticron | | | Fiberwire | | | | | | |
| Other, please specify: | | | | | | | | | | | | | | | | |
| **6. Your choice of core suture size.** | | | | | | | | | | | | | | | | |
| 2-0 | | 3-0 | | | | | 4-0 | | | 5-0 | | | | | | |
| Other, please specify: | | | | | | | | | | | | | | | | |
| **7. Your choice of peripheral repair technique.** | | | | | | | | | | | | | | | | |
| Simple-running | | Simple-locking | | | | | Cross-stitch | | | Hastled | | | | | | |
| Other, please specify: | | | | | | | | | | | | | | | | |
| **8. Your choice of peripheral suture material.** | | | | | | | | | | | | | | | | |
| Prolene | | Nylon | | | | | Ticron | | | Fiberwire | | | | | | |
| Other, please specify: | | | | | | | | | | | | | | | | |
| **9. Your choice of peripheral suture size.** | | | | | | | | | | | | | | | | |
| 5-0 | | 6-0 | | | | | 7-0 | | | 8-0 | | | | | | |
| Other, please specify: | | | | | | | | | | | | | | | | |
| **10. Your choice of postoperative rehabilitation protocol.** | | | | | | | | | | | | | | | | |
| Immobilisation | | | | | | Early passive mobilisation | | | | | | Early active mobilisation | | | | |
| Other, please specify: | | | | | | | | | | | | | | | | |
| **11. What is the average operation time for hand tendon repair?** | | | | | | | | | | | | | | | | |
| < 20 min. | | 20 to 39 min. | | | | | 40 to 59 min. | | | 60 to 79 min. | | | | | > 79 min. | |
| **12. What is the most common complication of flexor tendon repair?** | | | | | | | | | | | | | | | | |
| Re-rupture | | | | | | Infection | | | | | | Adhesion formation | | | | |
| Other, please specify: | | | | | | | | | | | | | | | | |
| **12.1 What is the approximate percentage of patients with the complication?** | | | | | | | | | | | | | | | | |
|  | | | | | | | | | | | | | | | | |
| **13. Are you interested in using endoscope and fibre optics in hand tendon repair?** | | | | | | | | | | | | | | | | |
| Very interested | | Interested | | | | | Neutral | | | Not interested | | | | | Very not interested | |
| **14. Endoscopic tendon retrieval method has potential to improve tendon repair outcome.** | | | | | | | | | | | | | | | | |
| Strongly agree | | Agree | | | | | Neutral | | | Disagree | | | | | Strongly disagree | |
| **15. Minimally invasive surgery can shorten tendon repair operation time.** | | | | | | | | | | | | | | | | |
| Strongly agree | | Agree | | | | | Neutral | | | Disagree | | | | | Strongly disagree | |
| **16. Biodegradable tendon repair device that degrades during tendon healing can improve tendon repair outcome.** | | | | | | | | | | | | | | | | |
| Strongly agree | | Agree | | | | | Neutral | | | Disagree | | | | | Strongly disagree | |
| **17. 3D printed tendon repair device can replace suture in hand tendon repair surgery.** | | | | | | | | | | | | | | | | |
| Strongly agree | | Agree | | | | | Neutral | | | Disagree | | | | | Strongly disagree | |
| **18. You would support a more expensive tendon retrieval and repair device if it led to reduced surgery time, improved recovery and outcomes.** | | | | | | | | | | | | | | | | |
| Strongly agree | | Agree | | | | | Neutral | | | Disagree | | | | | Strongly disagree | |
| **19. Any other comments about hand flexor tendon repair?** | | | | | | | | | | | | | | | | |
|  | | | | | | | | | | | | | | | | |
| **20. What is your gender** | | | | | | | | | | | | | | | | |
| Male | | | | | Female | | | | | | Other | | | | | |
| **21. What is your age** | | | | | | | | | | | | | | | | |
| 20 to 29 | 30 to 39 | | | | 40 to 49 | | | | 50 to 59 | | 60 to 69 | | | | | >69 |
| **22. What is your ethnic background?** | | | | | | | | | | | | | | | | |
| White | | | | Black | | | | | Mixed | | | | Asian | | | |
| Other, please specify: | | | | | | | | | | | | | | | | |
| **23. Number of years’ experience in hand specialty** | | | | | | | | | | | | | | | | |
| <3 | | | | 4 to 7 | | | | | 8 to 11 | | | | >11 | | | |
| **24. What is the type of your surgery?** | | | | | | | | | | | | | | | | |
| NHS | | | | | Private | | | | | | Both | | | | | |

Supplementary Table 2. Demographics of respondents.

| **Gender** | **Age** | **Ethnic background** | **Experience in hand specialty** | **Type of surgery** |
| --- | --- | --- | --- | --- |
| Male: 78% | 20 to 29: 1% | White: 73% | < 3 years: 7% | NHS: 70% |
| Female: 22% | 30 to 39: 30% | Asian: 24% | 4 to 7 years: 22% | Private: 4% |
|  | 40 to 49: 42% | Mixed: 2% | 8 to 11 years: 23% | Both: 26% |
|  | 50 to 59: 22% | Other: 1% | Over 11 years: 48% |  |
|  | 60 to 69: 4% | Black: 0% |  |  |
|  | Over 69: 1% |  |  |  |
